# Shaping of Curvilinear Steel Bar Structures for Variable Environmental Conditions Using Genetic Algorithms—Moving towards Sustainability

**DOI:** 10.3390/ma14051167

**Published:** 2021-03-02

**Authors:** Jolanta Dzwierzynska

**Affiliations:** Department of Architectural Design and Engineering Graphics, Rzeszow University of Technology, Al. Powstancow Warszawy 12, 35-959 Rzeszow, Poland; joladz@prz.edu.pl

**Keywords:** structural optimization, adaptive structure, steel design, parametric design, mechanical properties, sustainability, structural steel, multi criteria optimization, genetic algorithms

## Abstract

The successful and effective shaping of curvilinear steel bar structures is becoming an increasingly complex and difficult task, due to the growing demands to satisfy both economic and environmental requirements. However, computer software for algorithmic-aided design makes it possible to take into account many aspects affecting structures, as early as the initial design stage. In this context, the paper presents an optimization method for shaping the curvilinear steel bar canopies of hyperbolic paraboloid and cylindroid shapes, in order to obtain effective structures adapted to external environmental conditions. The best structural solutions in terms of the structure’s shape, topology and support positions are obtained as the effects of multi-criteria optimizations with the application of genetic algorithms. The following are used as the optimization criteria: minimal structure mass and minimal deflections of the structure’s members, as well as their maximal utilization. Additionally, the best canopy locations in relation to the sides of the world are determined through analyzing their shadow casts for various locations, so the structures have the least impact on the surroundings. This research, with its interdisciplinary character, aims to present the possibility of applying generative shaping tools to obtain structurally effective and environment-adaptive curvilinear steel bar structures in the first phase of their design, which can support sustainable designing.

## 1. Introduction

Steel as a structural material is used on a large scale for roof structures. Over the years, various methodologies and different methods have been developed for the shaping and designing of roof steel structures according to the current development of design trends and applied technologies. However, regardless of these factors, the process of shaping any steel structure has to be adapted to architectural and structural requirements, which are partly dependent on various environmental conditions. Meeting these requirements has become easier recently, as the architectural, engineering and construction industries have shown great improvements and cooperation in both theoretical and practical areas [1]. In general, any shaping method can be determined as the optimization of a structure’s shape, material and dimensions in order for it to meet the assumed criteria to the greatest extent. These are usually requirements of its strength and future use [2]. The requirements of the design standards for steel structures are related conditions regarding load-bearing capacity, serviceability, reliability, resistance to exceptional impacts of the natural environment, etc., which are interrelated [3,4,5,6,7]. The important issue is the design and calculation of the joints and nodes of steel bar structures, whose behaviour is often tested in experimental way [8,9]. This is the reason why the shaping of any steel structure should be considered in terms of various aspects, such as the structure’s function, safety, applied technology, and material solutions. Thus, the shaping criteria can be divided into the following groups: geometric–structural criteria, functional criteria, static strength and stability criteria, technological and material criteria, which are related to each other [10,11].

Due to the fact that the shaping stage is one of the first phases of the design, it is a very creative phase, but at the same time very important for the final structure’s form, which is expressed in references [12,13]. Therefore, at this stage, as many aspects as possible should be considered regarding the structure being shaped, in order to provide a reliable and efficient structure with an adaptable form and the minimum cost. Several potential structural design methodologies are illustrated in the literature, such as minimizing material consumption and production energy, analysis of life cycle, maximizing structural reuse, etc. [14].

Generally, curvilinear bar structures are defined as space structures made of thin bars that are connected in order to carry various loads. Curvilinear steel bar structures, mainly lattice structures of a cylindrical form, were developed in the mid-nineteenth century. However, due to certain problems both in their design and their construction from repetitive elements, they began to be applied on a large scale only in the 1940s. This time, the most popular were single-layer geodesic domes formed using the Buckminster Fuller procedure for dividing a sphere into triangles [15,16]. Therefore, the task of determining the best regular division of a spherical surface was one of the main challenges for scientists in the field of steel space structures [17]. Over the years, various approaches to the division of a sphere have been developed in order to obtain different types of structures, such as Schwedler’s and Lamell’s lattices [17]. However, combining various elements of the achieved lattices into larger models was one of the first methods of designing new shapes of curvilinear steel bar structures. A broad overview of the different kinds of spatial mesh structures, their improvement, and their applications, is presented in reference [18]. However, various analytical approaches to shaping double-layer trusses are shown in reference [19], whereas the method of shaping steel bar structures by locating their nodes on the so-called base surfaces is presented in reference [20,21,22,23,24]. Nowadays, thanks to advancements in technology, curvilinear steel bar structures with a great variety of geometries and technical solutions are being developed on a large scale. These structures can be mostly used as load-bearing roof structures, as well as both vertical and horizontal divisions of various building spaces. Due to bar structures’ high strength to weight ratio, as well as due to their great aesthetic values, their shaping is worth analyzing.

The method of the geometric modeling of curvilinear steel bar structures depends mostly on the properties of the base shell used for forming the structure. These properties are important as the type of the base surface applied determines the static properties of the obtained grid structure, as well as its topology. Therefore, in the case study presented in the paper, ruled surfaces are used as base surfaces for a hyperbolic paraboloid and a cylindroid. These ruled base surfaces are composed from straight lines, which is advantageous due to their discretization. It is worth noting that the hyperbolic paraboloid provides a particularly interesting basic shape for a variety of single or complex building roof forms [24]. This shape was introduced, and found the use in the construction of thin shells, in the post-war era through the development and combination of contemporary architecture and structural engineering. The hyperbolic paraboloid, due to its double curvature, is extremely rigid. It exhibits a membrane effect, so internal forces are effectively transferred by its surface. It is the focus of various publications, which deal mostly with theoretical, experimental and design aspects of hyperbolic paraboloid concrete or reinforced concrete structures [24,25,26]. However, an analysis of the gabled hyperbolic paraboloid shell is performed in reference [25]. Due to hyperbolic paraboloid’s positive static properties, they have found application in creating lightweight shell concrete structures with large spans, and have also been used to form complex multi-shell structures from single ones [26,27], which was pioneered by F. Candela. Despite the widespread application of the hyperbolic paraboloid surface for roof construction, there is still little research being done on the influence of its divisions, or the consequentially obtained grid patterns, on the load-bearing capacity of the steel bar structures being formed [22]. However, as far as the roofs of cylindroid shapes are concerned, they are not as popular as the hyperbolic paraboloid ones and they receive little attention in the literature.

An important issue for shaping curvilinear steel bar structures is establishing an appropriate structural shape, a supporting system, and the topology, that is, the bars’ positions in the structure, as well as their cross-sections. The topology is partly determined by the overall geometric concept of the structure, and partly by the general criteria of rational shaping. There are two main shaping methods mostly used to describe the positions of bars in regular steel bar structures, that is, numeric and geometric. The first method uses a grid of points and their coordinates, as well as the so-called direction vectors, in order to define bar structures. The second one defines the so-called basic solids that both fill and divide the given space in order to form bar structures [12]. However, the basic principles of the geometrical shaping of structures is described in the literature on the subject, which ensure both the efficiency and the reliability of the structure are the principles of its regularity and symmetry. Moreover, the principle of the minimization of construction eccentricities that can generate additional torsional or bending moments is important too [28].

The method of shaping structures depends also on the design tools used, the applicability of which changes with the development of technology [29]. Commonly used CAD (computer-aided design) tools enable the generating of virtual models, their visualization, as well as the creation of their two-dimensional documentation [30,31,32]. However, the further development of other digital design tools, as well as the integration of various modeling methods, has facilitated and simplified cooperation in various design fields [33,34,35]. Such an interdisciplinary approach is especially needed for shaping curvilinear steel bar structures, the design of which involves many different issues and sometimes can be a great challenge. The development of modern software tools equipped with visual scripting languages, such as the Rhinoceros 3D software, has significantly influenced the designing process both in architectural engineering and in civil engineering [1,36]. Thanks to these tools, virtual and spatial forms of designed structures are described by means of advanced algorithms based on parametric equations. This enables the generating and modifying of the digital models of original spatial structures, as well as helping to adjust them for various environmental conditions [37,38]. Parametric tools are especially useful in the conceptual phase of design, which deals with the multi-variant and often interdisciplinary analysis of initial solutions. The algorithmic-aided shaping of steel bar structures is a novel area of research. However, an attempt to design steel structures with the use of algorithms was undertaken in references [20,21,22,23], whereas a similar shaping of concrete shell structures was presented in reference [27].

Nowadays, there is a growing interest in the application of EO in structural engineering. Some of the research explores the possibility of using genetic algorithms to optimize the structures being designed. The method using genetic algorithms for designing non-linear steel frames with the application of semi-rigid connections is presented in reference [39]. Thanks to the application of algorithms for selecting suitable cross-sections from a standardized set of steel cross-sections, the authors obtained a minimum-weight frame. The structural topology optimization of single layer grid structures is given in reference [40], whereas a three-dimensional topology optimization method for achieving hollow structures, applying moving morphable components, is presented in reference [41]. Algorithmic-aided structural optimization can be performed using both gradient and non-gradient algorithms. An explanation of this issue is presented with an example of the truss size optimization problem in reference [42]. Open-source and benchmarking software for helping one to understand the process of structural optimization is provided there. Truss optimization with frequency constrains, and the application of a hybrid optimality criterion and a genetic algorithm method, are presented in references [43]. It is shown that this hybrid method has great potential in searching for optimal truss structures, as well as it requires less computational work. The optimization of the cross-sectional shapes of thin-walled beams of automobile structures using genetic algorithms is presented in reference [44]. The area of the cross-section is established as an objective function with various constraints, such as cross-sectional stiffness, manufacturability, and cost. An efficient algorithm for the layout optimization of truss structures is proposed in reference [45]. On the other hand, shaping structures in an optimal way, taking into account some environmental aspects, is shown in reference [21].

The goal of this research is to elaborate on the algorithms in order to create digital models of curvilinear steel bar canopies of cylindroid and hyperbolic paraboloid shapes with various grid patterns. Further, it aims at an evaluation of the load-bearing capacity of these curvilinear steel grid structures, dependently on their curvatures, the supporting systems and the topologies. Its goal is also to show how the properties of the structural material largely determine the behaviour of the structure during optimization, and have an impact on finding its final and optimal form. Particularly, the article attempts to perform ESO (multi-objective evolutionary structural optimization) on the considered canopies with respect to structural requirements, which means obtaining effective structures, the reduction of steel consumption, and finding a sustainable design. A similar approach was presented in reference [22]; however, this study performed only two-objective evolutionary structural optimization, and the considered hyperbolic paraboloid structure was supported by four single columns. This research also aims to compare the efficiencies of curvilinear steel bar structures of hyperbolic paraboloid and cylindroid shapes, and aims to find the most effective and optimized structure. Additionally, the canopies’ best locations in relation to the sides of the world are determined through analyzing their shadows cast during the considered period, in order to determine the impact of the structure on its surroundings.

In a broader sense, this paper is intended to show how to apply generative tools to integrate geometry and structural efficiency as a single goal, in order to obtain structurally effective and environment-adaptive steel roof structures in the first phase of their design. The reason for choosing such a topic was driven by the great potential of parametric and optimization methods for shaping rationalized steel bar structures.

## 2. Materials and Methods

The shaping of any curvilinear steel bar structure, associated with giving it the most appropriate and optimal form, can be treated as the successive specification of the parameters that describe it. Contemporary tools currently on the market for both geometric modeling and structural analyses require less effort and computing time, but most of them work in a single-track mode, as separate tools are used to generate the complex geometry, and to perform the structural calculations. However, modern tools for the parametric shaping of structures, which work in the Rhinoceros 3D environment (Rhino 6, ProGrupa, Poznan, Poland) created by Robert McNeel and associates, give new possibilities for shaping [46]. Rhinoceros 3D is a modeling software enabling the creation of various free-form shapes based on non-uniform rational b-splines (NURBS). However, the Grasshopper plug-in, built in Rhinoceros 3D makes it possible to create scripts defining parametric models. These models can be further analyzed and modified, as well as visualized, in a Rhinoceros’s viewport.

The shaping strategy with regard to curvilinear steel bar structures undertaken during the research consisted of creating curvilinear steel bar grid structures by locating the structures’ nodes on the so-called base surfaces, being cylindroids or hyperbolic paraboloids. Due to the fact that the structures’ geometries, dimensions and grid topologies were defined parametrically, the first step was the elaboration of the universal scripts of the parametric digital models of various canopies with different bar grid topologies and supporting systems.

Grasshopper algorithmic modeling is a tool representing a graphical programming language integrated with Rhinoceros 3D. Each Grasshopper parametric script consists of various graphical block components, which are placed on the canvas. These components need to be connected logically in order to perform various actions. Moreover, the components need input data to act and produce output information as the action is carried out. Initially, Grasshopper was mostly applied for parametric modeling in architecture and fabrication problems. However, thanks to its capabilities and thanks to enriching it with new plug-ins, it has begun to be applied in structural engineering [1,42,46,47]. The Grasshopper parametric scripts created for the presented research defined variable shapes, dimensions and topologies of the structures being shaped, in order to explore their forms. Next, the scripts defining the geometric forms were converted into the scripts defining structural models by means of a Karamba 3D educational plug-in [46,48]. This enabled us to analyze, according to Eurocode 3, the parametric structural models of various geometrical forms with different topologies, subjected to various loads.

Finally, the performed scripts made it possible to simulate and optimize the structural models with the application of evolutionary structural optimization (ESO), which was performed by other Grasshopper’s plug in, that is Octopus 3.4 [49]. Evolutionary optimization (EO), genetic optimization is based on a genetic algorithm inspired by the Darwinian law of natural selection. The genetic optimization process mimics the natural processes of genetic coding, selection and reproduction, in order to iteratively improve the individuals in each generation, according to the optimization criteria related to fitness functions. EO finds the optimal solution in a population of possible variables, rather than improving a single variable.

In the presented research, following the performed simulation, the structures’ support positions were optimized by taking into account the minimal structure masses, the minimal deflections under self-loads, and the maximum utilization of structural members. The solving of multi-objective optimization problems consists of choosing an optimal solution from the so-called Pareto front, which visualizes possible solutions with trade-offs between the different criteria. This further allowed us to evaluate various alternatives to of the considered structures, and to choose optimal design solutions that meet the established criteria. Next, we performed structural analysis for the best solutions, and the structures’ members were optimized by means of the Robot Structural Analysis Professional 2019 software (Autodesk) [50], taking into account environmental loads acting on the structures. As the optimization criteria, the minimal masses of the structures were considered. Finally, the canopies’ best locations in relation to the sides of the world were determined through analyzing their shadows cast during the established period in various locations using the Ladybug 0.0.66 plug-in [51], such that the structures had the least impact on their surroundings.

## 3. Results

As was mentioned previously, the shaping of steel bar structures is treated as solving a problem with all the requirements and constrains it presents. Parametric shaping is an action aimed at choosing the values of some structural parameters that meet the shaping criteria. The shaping criteria can be different; however, for steel structures, the main goal is the reduction of the structures’ mass, structural cost minimization and the unification of the structures’ elements, as well as the application of uncomplicated joints. For most design-optimization problems, the following formulation procedures are used: problem statement; collection of data; definition of shaping variables; identification of optimization criteria; identification of constrains. However, the general scheme applied for the shaping of the considered curvilinear steel bar structures was as follows: geometric modeling of the structures; establishing their structural models; performing the multi-objective optimization of the structures under a dead load; selecting the best solutions; performing the structural analysis of these, taking into account various load combinations.

### 3.1. Geometric Modeling of the Structures

**Figure 1:**
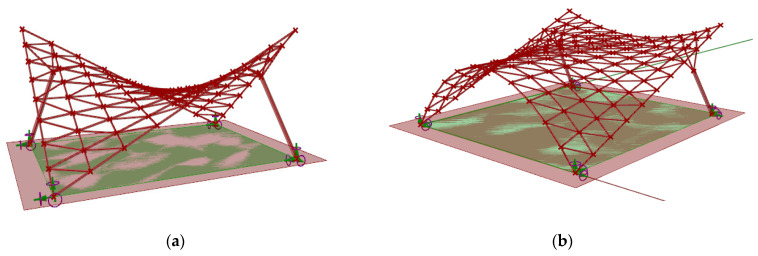
The view of the considered structures of: (**a**) hyperbolic paraboloid shape; (**b**) cylindroid shape.

The geometric modeling approach used during the research concerned canopy roofs of a hyperbolic paraboloid shape (number 1) and of a cylindroid shape (number 2) (Figure 1). For the conducted analysis it has been assumed that every roof structure covers a rectangular area of one hundred square meters. Moreover, each structure was supported by four supports, two of which were attached directly to the grid structure and were placed on the ground in two corners of the rectangular place (ground supports), whereas, the other ones were the bases of the columns supporting the structure (column supports). The columns applied were perpendicular to the base surface of the roof and were attached to the structure by four bar branches connecting them with the nearest lattice’s vertices.

**Figure 2:**
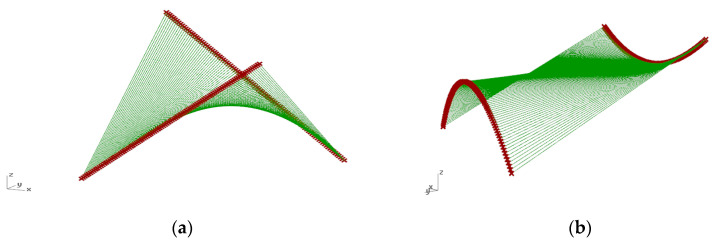
The base surfaces for bar grids of: (**a**) hyperbolic paraboloid shape; (**b**) cylindroid shape.

Both the hyperbolic paraboloid and the cylindroid are ruled surfaces composed of straight lines—the so-called rulings. Each surface can be defined by two directrices, which are lines, and a so-called director plane to which all rulings are parallel. However, the hyperbolic paraboloid roof surface can be established by two screw lines, whereas the cylindroid roof surface can be established by two curved lines included in various planes (Figure 2). In the presented study, the cylindroid was determined by two parabolas shaped in opposite directions and included in the parallel planes.

**Figure 3:**
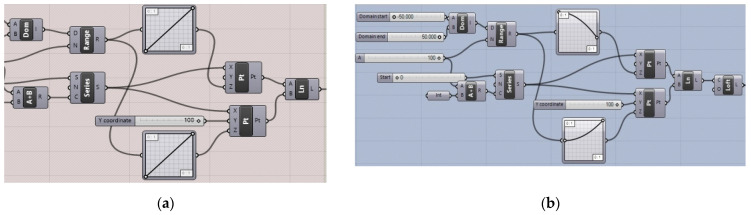
Grasshopper scrips for the definition of: (**a**) the hyperbolic paraboloid and (**b**) the cylindroid.

The surfaces have been defined parametrically by means of Grasshopper’s block scripts, presented in Figure 3, and have been established by the following procedure: each directrix line was divided into the same number of parts; next, each surface ruler was defined by connecting two proper points that are included in various directrix lines.

**Figure 4:**
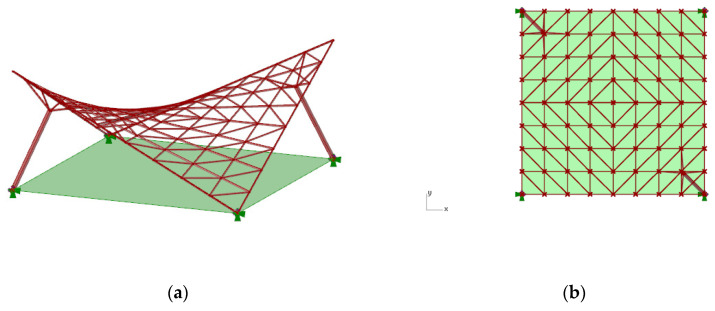
The hyperbolic paraboloid structure of type a (with a grid division along shorter diagonals): (**a**) an axonometric view; (**b**) an orthographic projection.

**Figure 5:**
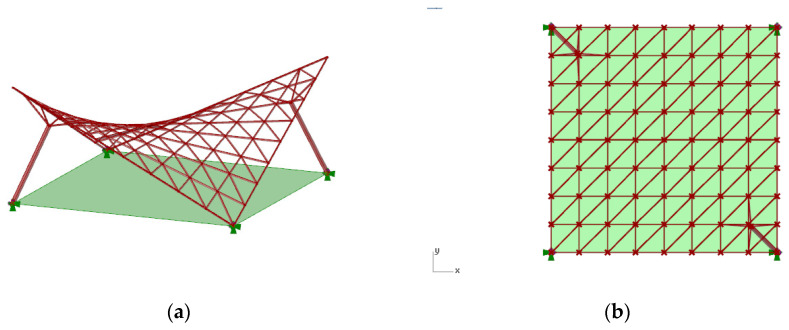
The hyperbolic paraboloid structure of type **b** (with a grid division along longer diagonals): (**a**) an axonometric view; (**b**) an orthographic projection.

However, the base surfaces were defined as sets of rulers, and further discretized by division into the same number of parts in both *x* and *y* directions. In this way, grid vertices were established as the points placed on the base surfaces. In general, surface discretization, that is, the process of its division into units, can be done in various ways. The possible methods of surface discretization differ depending on the number of faces applied, their curvature and shape, as well as the number and complexity of the nodes. In the presented case studies, planar faces (panels) were applied, which are always preferred to curved panels due to the production cost. In order to achieve flat panels, each of the obtained spatial polygons was divided into two triangles. As a result of such discretization, two kinds of triangular bar grids with different patterns were obtained. This was due to the fact that every grid quadrangle can be divided along both shorter and longer diagonals. Depending on the kinds of the divisions applied, the structures are named as follows: the structures with the division along the shorter diagonals are structures of type a (Figure 4), and the structures with the division along the longer diagonals are structures of type b (Figure 5).

It is worth noting that such divisions can define various structures whose grid patterns depend on the number of base surface subdivisions applied.

In the presented case study, the positions of the ground supports were fixed, whereas the best positions of the column supports were established following the structure’s performance. We have additionally assumed that the columns are not placed further than one meter from the place’s border, both inside and outside of it. The columns’ positions were defined parametrically by coordinates *x* and *y*, determined on the *x* and *y* axes, with the units used equal to 1m. For both a hyperbolic paraboloid shape and a cylindroid shape, all structural members were established as members with round hollow cross-sections. However, as the roof coverings, glass panels have been proposed.

### 3.2. Optimisation of the Structures

**Figure 6:**
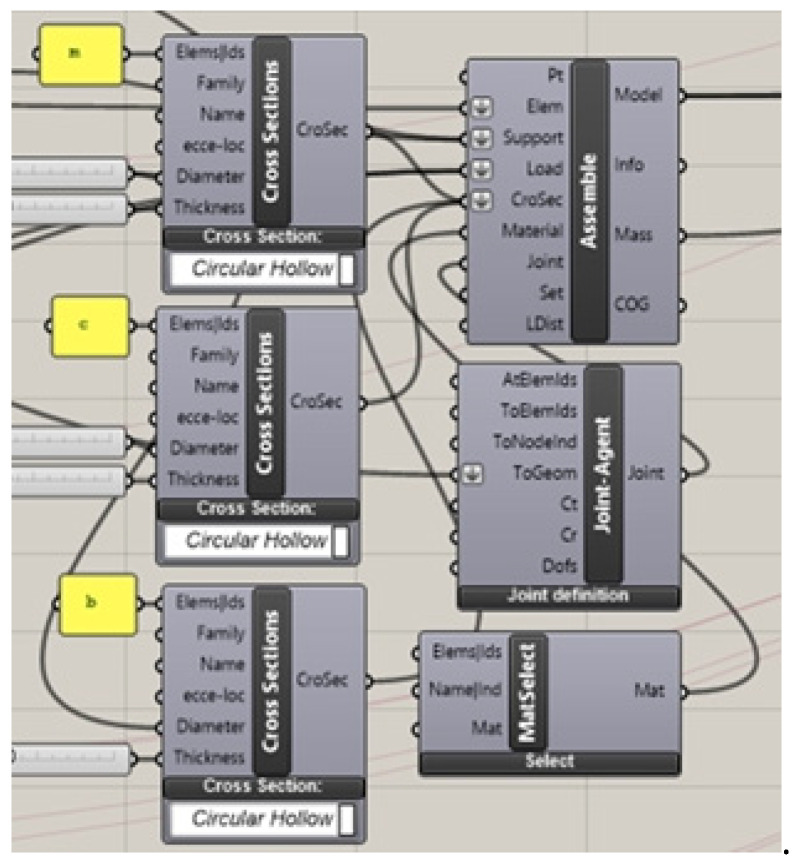
The fragment of the Karamba 3D script for structural analysis.

In order to optimize the obtained structures, the scripts determining the geometrical characteristics of the curvilinear steel bar structures were developed by means of the Karamba 3D plug-in. Due to this fact, the bar grids’ vertices were transformed into structural nodes. They were also established structural joints: rigid for lattice and hint joints, which connect the lattices with branches. The fragment of the Karamba 3D script is presented in Figure 6.

The first step in the conducted analysis was the determination of the supports’ positions by means of EO, which was the multi-criteria optimization performed by Grasshopper’s plug-in Octopus. Octopus allows for searching for many goals at once by producing a range of optimized trade-off solutions between the established extremes of each goal. However, the optimization was used to evaluate the structures’ behaviors under self-loads, in order to establish proper support locations, members’ cross-sections, as well as proper grid topologies. In Octopus optimization, the objective function is hidden. Everything is designed by block components. The variables and optimization goals are entered into the special component, and if everything works well in the script, the optimization runs. The optimization of each structure has been carried out with the following objectives:

- minimize the structure’s mass;
- minimize deflection;
- maximize elements’ utilization.

However, the variables for optimization were as follows:

- height of the structure;
- locations of supports;
- lengths of lattice bars;
- locations of common nodes of each group of column branches.

The additional data assumed for the optimization, that is, the structural constraints and fixed variables, are listed in the following points:

- compliance with ultimate limit states and serviceable limit states;
- kinds of cross-sections, both for lattice and columns (circular hollow);
- dead weight as the sum of the structure’s weight and the panels’ weight, calculated by Karamba 3D for each roof individually;
- number of supports—four (two of them fixed);
- structural material (steel S235);
- panel material—glass with density of 2400 kg/m³ and thickness of 6 mm.

Range of variables to be optimized:

- height of the structure (from 4 to 6 m);
- location of two supports—within the considered place, but no further than 1 meter from the place’s border, both inside and outside of it;
- diameter of the cross-sections (from 4 to 6 cm for both lattice bars and branches, and from 5 to 8 cm for columns);
- minimal bar length (from 1 to 1.5 m);
- maximum bar length (from 1.5 to 2.0 m);
- the location of the common node of each group of column branches within the distance of 0.5 to 1 m from the lattice surface.

Additionally, the following parameters were assumed for the simulation:

- a population size of 100;
- mutation probability equal to 0.1;
- mutation rate equal to 0.5;
- crossover rate equal to 0.8.

**Figure 7:**
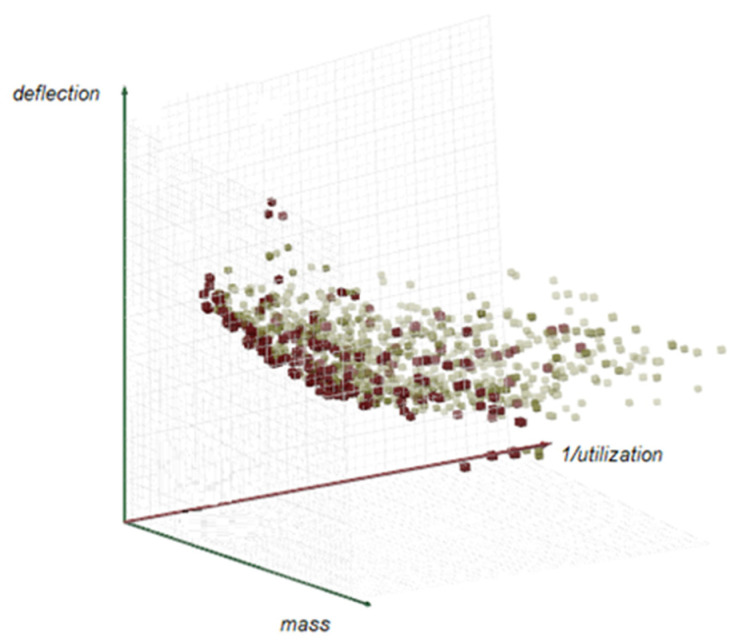
3D Pareto front received during optimization.

Via the performed optimization, the elements’ cross-sections have been pre-determined. However, in order to make it easier to compare individual structures, a second optimization was performed assuming the same minimal typical cross-sections of the structural members, and also taking into account the constraints that had been established earlier. Given the optimization goals expressed previously, that, is the total mass minimization, the minimization of deflections, and the maximization of the utilization of bars that are conflicting, four results from the Pareto front for each structure with pattern **a** and **b** have been chosen. The Pareto front for hyperbolic paraboloid optimization is presented in Figure 7. In the case of each multi-objective optimization with three objectives, the Pareto front is a 3D surface composed of the points that are the results of optimization, which are the best compromises. However, each result includes the established values of the parameters of mass, deflection and utilization.

These were considered as good trade-off solutions. The results of the optimization with three optimization objectives (minimal total mass, minimal deflection, maximum utilization) mentioned earlier are given in Table 1 and Table 2.

The simulation results for the hyperbolic paraboloid structure of type **a** are shown in Table 1, whereas the simulation results for the hyperbolic paraboloid structure of type b are shown in Table 2. The optimal height of the structure was established as 5 m.

However, the simulation results for the cylindroid structure of type **a** are shown in Table 3, whereas the simulation results for the cylindroid structure of type **b** are shown in Table 4.

Analyzing the above results of the optimization, it can be stated that the cylindroid canopy structure is much heavier than the hyperbolic paraboloid canopy structure covering the same plan. With the diameter and the thickness of the cross-sections proposed, the maximum nodal displacement of the cylindroid canopy roof is equal or close to the admissible value of 40 mm. Due to this fact, the hyperbolic paraboloid canopy structure was taken for further consideration as the more economic structure.

However, after analyzing the results for the hyperbolic paraboloid roof presented in Table 1 and Table 2, it can be stated that individuals a1, a3, b1 and b3 with established column positions are the most efficient. Due to this fact they were considered further.

The next step was the verification of statics, ultimate limit states and serviceable limit states, considering various load combinations of both self-load and environmental loads (snow and wind) for the chosen individuals. Therefore, the structures a1, b1, a3 and b3 have been analyzed by means of the Robot Structural Analysis Professional software. The starting assumptions for the conducted calculations are presented below; some of them were established by the first simulation.

Starting assumptions for each of the calculations:

- height of the structure—5 m (received from the first simulation);
- kind of cross-sections—circular hollows with thickness of walls no less than 3.2 mm;
- lattice’s division—along the axis *x* into eight parts and along the axis *y* into eight parts (received from the first simulation);
- locations of supports according to the coordinates established earlier (received from the first simulation);
- locations of the common nodes of each group of branches—0.7 m from the roof’s surface (received from the first simulation);
- assumed location of the canopy structure—Rzeszow, Poland.

The structures were optimized via the worst-case scenario resulting from the combination of loads. As the minimization objective, the minimum structural mass was taken. The analysis showed that the considered structures were mostly loaded by normal forces, whereas the bending moments were minimal. However, due to the uneven distribution of stresses in the grid’s bars, the bars have been divided into three groups as follows: internal grid bars, external grid bars, support grid bars. The grid’s support bars were the bars at the supports, which were the most loaded. These were arranged as six external lattice bars at each ground support in the case of the structure with grid pattern **a,** and five bars (four external grid bars and one internal grid bar) at each ground support in the case of the structure with grid pattern **b**. Assuming the division of the roof lattice into eight parts in two directions, all structures were composed of 218 bars: two columns, eight branches and 208 lattice bars.

The results of the structure dimensioning and member verification for four individuals (a1, a3, b1, b3) obtained with the Robot Structural Analysis Professional software are presented in Table A1. This analysis has shown that the individuals with a grid pattern of type b are more effective due to their masses. However, it can be concluded that the structures with a grid pattern of type a are more efficient, when comparing the diversity of the assortment of bars applied in both types of structures (**a** and **b**).

**Figure 8:**
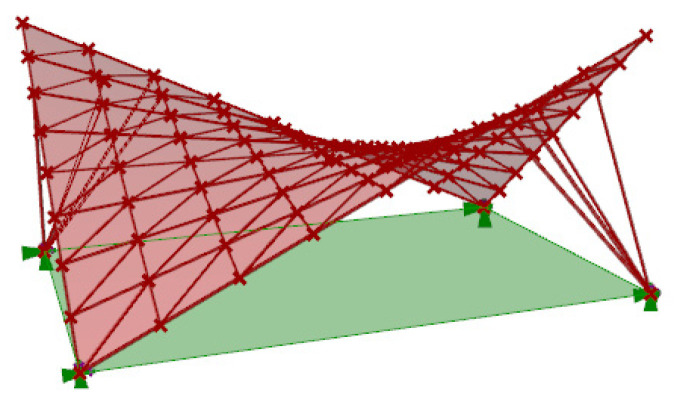
The hyperbolic paraboloid roof with branches supported on the ground.

The subject of another analysis was to compare the effectiveness of the considered structures in the case of using bunches of columns instead of single columns (Figure 8). For this purpose, the created algorithms were used for simulation; however, the assumed columns’ lengths were equal to zero.

With the same border conditions applied as in the cases considered previously, the mentioned structure has been dimensioned according to stress distribution in the way presented in Table A2. Based on the results regarding the masses of structures 1a and 1b, shown in Table A1 and Table A2, it can be stated that the canopy roof, in which bunches of columns were used instead of single columns, is lighter than the same roof supported by single columns.

As the mass of the bar structure largely depends on the density of the bar mesh used, the aim of the research was also to check the change in mass of the structure in the case of a reduction in the density of the bar meshes. Therefore, the following simulation and optimization of a similar hyperbolic paraboloid roof structures was made, assuming longer grid bar lengths, as follows:

- minimal bars’ length—from 1.5 to 2 m;
- maximum bars’ length—from 2 to 3 m.

**Figure 9:**
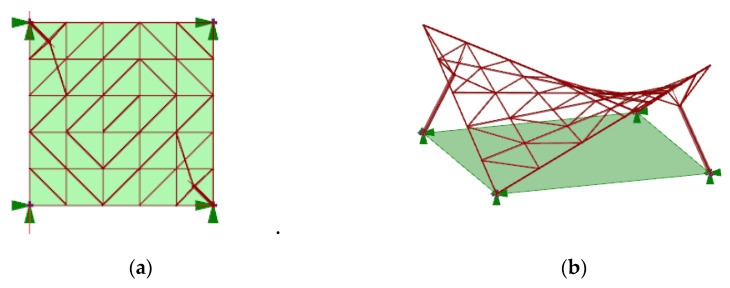
The structure of type **a** with the division of the grid pattern into five units: (**a**) an orthogonal projection; (**b**) an axonometric view of the structure.

The analysis of the hyperbolic paraboloid canopy roof structures **a** and **b**, with smaller divisions in both the *x* and *y* directions than in the previous cases, has shown that more efficient structures can be achieved due to their masses, as well as due to a number of the structural members applied. The division into five parts in both directions allows a large reduction in the numbers of bars and vertices. However, the best optimization result, that is, the least variety of bars, as well as a relatively small mass of the structure, was obtained for the structure with pattern **a**, shown in Figure 9.

The grid of the mentioned structure is composed of five members, whereas the grid of a similar structure, with a grid pattern of type **b**, is composed of seven members. The bar characteristics of the optimized structure are given in Table 5. As from a technological point of view, a grid composed of square pipes is more convenient for glazing than one composed of round pipes, solutions have been given for these two types of cross-sections (Table 5). It is worth mentioning that the structure composed of square pipes is lighter than the other one.

The cross-sections of the structural elements presented in Table 5 vary greatly. In order to reach a uniform structure, the bars should be standardized by reducing the diameter and increasing the wall thickness of the support bars. However, a good solution would be the use of joints in the system that enable the connection of bars of various diameters. Analyzing the results of the conducted simulations and optimizations, it can be stated that the structure divided into five segments in both directions and with pattern **a**, and also with columns set in the corners of the square, can be chosen as the best solution within the established criteria.

### 3.3. Analysis of the Shadows Cast by the Shaped Structures Dependent on their Locations

**Figure 10:**
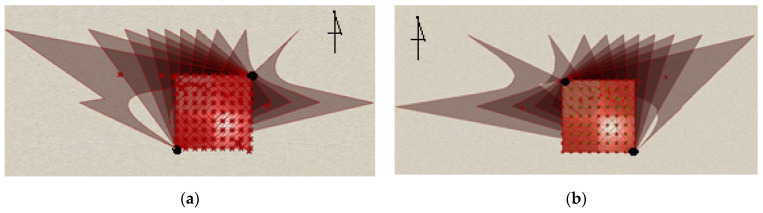
The border of the shadow cast depending on the location of ground supports: (**a**) a right ground support on the north side; (**b**) a left ground support on the north side.

The last element of the study was the determination of the size and the border of the shadows produced by the canopies, depending on their locations with respect to north. Two positions for the structures have been considered, differing in terms of the location of the ground supports (Figure 10). The analysis has been carried out using Ladybug, the Grasshoppers plug-in. During analysis, a period from March to September and between 10 a.m. and 5 p.m. was considered, and the structures’ locations were as determined previously, that is, Rzeszow, Poland.

Two positions for the structures within the given plan were considered: with a right ground support on the north side, and with a left ground support on the north side (Figure 10).

The analysis showed that a similar amount of shadow is produced in both structure positions, although it is slightly greater in the case of the position presented in Figure 10b. However, the extents of the shadows’ influences over the adjacent place are different, which may have an impact on the design of the structure’s location, unless there are other constraints.

## 4. Discussion

An original algorithmic-aided method of shaping curvilinear canopies of hyperbolic paraboloid and cylindroid shapes has been proposed. This way of shaping verifies canopies’ geometry with respect to structural requirements. For this purpose, we have elaborated effective procedures for the generative shaping of curvilinear steel bar structures, consisting of forming bar grids via the location of grid nodes on base surfaces that are cylindroids and hyperbolic paraboloids. Moreover, a transformation has been carried out of the principles of structural shaping, into logical–geometric and mechanical dependencies, by developing universal algorithms describing both geometrical and structural models of bar structures.

The method of shaping structures defined in this way is very efficient due to the possibility of using the potential of the computer program Rhinoceros 3D, as well as its various plug-ins, to create bar structures. However, in order to obtain effective curvilinear steel bar structures, it is necessary, as shown in the study, to select as base surfaces the surfaces with favorable geometrical and mechanical properties. The conducted analysis has shown that of the two considered types of bar structures formed on the basis of the hyperbolic paraboloid and the cylindroid, the former are more effective. The roof of the cylindroid shape with the optimal location of supports proved to be much heavier than the hyperbolic paraboloid roof. Due to the fact that the structure’s mass is one of the factors that influences its cost, it can be concluded that the hyperbolic paraboloid canopy is more efficient than the cylindroid one.

Although the shape of the hyperbolic paraboloid is widely used for roof-forming, our research on the structural efficiency of bar structures depending on the surface division in order to create structures is original. A multi-criteria analysis with the use of three objectives was applied, which is a new approach in the case of shaping curvilinear steel bar structures. The performed analysis of shaping curvilinear steel bar structures based on both the hyperbolic paraboloid and cylindroid has not only proved that the selection of a proper grid pattern has a significant impact on the efficiency of the structure, but it has also shown the method of creating the grid, if the new grid bar pattern results from the division of the previously given grid pattern. Triangular bar grids of curvilinear steel bar structures were created, dividing the spatial quadrilateral bar grids. This division took place along both the longer diagonals of quadrangles and the shorter diagonals. The study has shown big differences when it comes to the weight of the structure, depending on whether the bar quadrangles were divided along the shorter or longer diagonals. In the case of the studied models of the steel structures, the structures with the grid pattern split along longer diagonals were more efficient, as far as the structures’ masses are concerned. However, due to the diversity of the assortment of the structural members, the structures with the grid pattern split along shorter diagonals were more efficient. Unifying the assortment of bars is very important for every steel bar structure. This is not an easy task if the structure is formed in such a way that the structural nodes are placed on the base surface. In turn, the location of grid nodes on the base surface with favorable mechanical properties has a beneficial effect due to the distribution of forces in the bars, thanks to which cross-sections of the bars can be obtained.

The research has shown that multi-criteria optimization can help in determining the optimal positions of supports and the form of the structure. It has also proved that the structures supported by bunches of columns are more effective, due to their masses, than the structures supported by single columns. Moreover, the density of the bar meshes used during the studies had a significant impact on the weight of the structure. Multi-criteria optimization does not give a single unequivocal and optimal result, particularly when the optimization criteria are contradictory, as in the presented study. However, it does allow for an initial assessment of the behavior of the structure under environmental conditions, as well as does allow one to estimate possible alternatives, choose the right solution, and take the appropriate decision.

**Figure 11:**
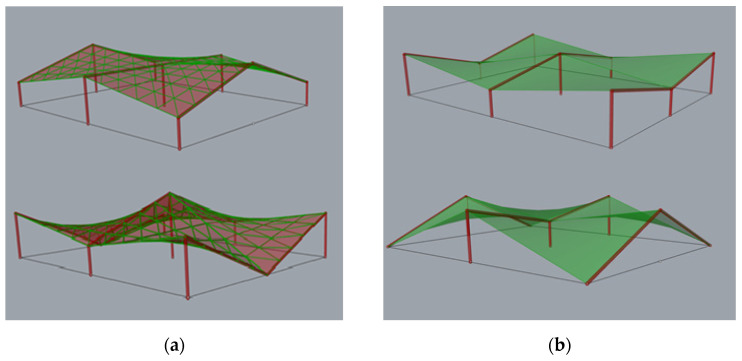
Examples of multi-segment structures composed of hyperbolic paraboloid units (**a**) with lattice roof and (**b**) with steel sheets roofing.

The developed method can be used in the case of shaping more complex, multi-segment structures, both with lattice roofs as well as with roofs composed of corrugated steel sheets, which will be the subject of further studies by the author (Figure 11).

Moreover, this research should be continued in order to consider various optimization objectives, such as the unification of members and the complexity of joints.

## 5. Conclusions

Over the years, the development of computer technology has meant that structural engineers have tools that require a different view and approach to the shaping of steel bar structures. Modern tools, such as the computer program Rhinoceros 3D and its plug-ins, enable parametric shaping, linking their geometry with structural analysis and optimization. In practice, any design problem has multiple competing objectives, regarding engineering, architectural and economic aspects, which are often conflicting and result in many possible solutions. As was shown in the study, in this case, so-called multi objective optimization can be applied.

The research developed original procedures for the effective algorithmic-aided shaping of curvilinear steel bar structures, such as hyperbolic paraboloids and cylindroids. It utilizes multi-criteria optimization, taking into account mechanical aspects, the simplicity of structures and the material consumption. The study has proven that the algorithm-aided shaping of steel bar structures with various shapes and topologies deserves attention, as it can be helpful in obtaining rational structures. By minimizing the weight of the structure, steel consumption is reduced, and the design is made greener. As was shown in the study, the impact of the structure on the surroundings can be taken into account as early as the initial stage of design, which may affect the location of the structure. Moreover, considering the interdisciplinary nature of design, the shaping of steel bar structures can improve a structure’s performance, and can be a guarantee of achieving eco-friendly structures as well as sustainable design.

## Figures and Tables

**Table 1:** The simulation results for the hyperbolic paraboloid structure with a grid pattern of type **a** (the grid split along shorter diagonals).

Number of the Structure	Position of Column 1 in Coordinates *x, y*	Position of Column 2 in Coordinates *x, y*	Mass (kg)/Maximum Displacement (mm)
a1	x_1_ = −0.4	x_2_ = 9.8	994.71/
	y_1_ = 9.8	y_2_ = −0.4	8
a2	x_1_ = −0.3	x_2_ = 10.3	1003.86/
	y_1_ = 10.3	y_2_ = −0.3	8
a3	x_1_ = 0.0	x_2_ = 10.0	1002.54/
	y_1_ = 10.0	y_2_ = 0.0	8
a4	x_1_ = −0.2	x_2_ = 10.2	1003.32/
	y_1_ = 10.2	y_2_ = −0.2	9

**Table 2:** The simulation results for the hyperbolic paraboloid structure with a grid pattern of type **b** (the grid split along longer diagonals).

Number of the Structure	Position of Column 1 in Coordinates *x, y*	Position of Column 2 in Coordinates *x, y*	Mass (kg)/Maximum Displacement (mm)
b1	x_1_ = −0.4	x_2_ = 9.8	1005.06/
	y_1_ = 9.8	y_2_ = −0.4	14
b2	x_1_ = −0.2	x_2_ = 9.8	1005.86/
	y_1_ = 9.8	y_2_ = −0.2	15
b3	x_1_ = 0.0	x_2_ = 10.0	1012.89/
	y_1_ = 10.0	y_2_ = 0.0	7
b4	x_1_ = −0.2	x_2_ = 10.2	1013.68/
	y_1_ = 10.2	y_2_ = −0.2	7

**Table 3:** The simulation results for the cylindroid structure with a grid pattern of type **a** (the grid split along shorter diagonals).

Number of the Structure	Position of Column 1 in Coordinates *x, y*	Position of Column 2 in Coordinates *x, y*	Mass (kg)/Maximum Displacement (mm)
a1	x_1_ = −0.0	x_2_ = 10.0	1801.34/
	y_1_ = 10.0	y_2_ = 10.0	40
a2	x_1_ = −0.2	x_2_ = 9.5	1802.09/
	y_1_ =10.3	y_2_ = −0.3	30
a3	x_1_ = 0.2	x_2_ = 10.0	1801.02/
	y_1_ = 9.8	y_2_ = 10.0	40
a4	x_1_ = −0.2	x_2_ = 9.8	2128.51/
	y_1_ = 9.8	y_2_ = 9.8	40

**Table 4:** The simulation results for the cylindroid structure with a grid pattern of type **b** (the grid split along longer diagonals).

Number of the Structure	Position of Column 1 in Coordinates *x, y*	Position of Column 2 in Coordinates *x, y*	Mass (kg)/Maximum Displacement (mm)
b1	x_1_ = 0.0	x_2_ = 10.0	1833.26/
	y_1_ = 10.0	y_2_ = 10.0	39
b2	x_1_ = 0.2	x_2_ = 9.8	1802.09/
	y_1_ = 10.0	y_2_ = 10.0	30
b3	x_1_ = 0.05	x_2_ = 9.6	2616.55/
	y_1_ = 9.5	y_2_ = 9.6	40
b4	x_1_ = 0.2	x_2_ = 9.8	2618.554/
	y_1_ = 9.8	y_2_ = 9.8	40

**Table 5:** The optimization results for the hyperbolic paraboloid canopy roof with the grid divided into five parts.

Kind of the Structural Element	Quantity/Length (m)	Total Quantity	Bars’ Cross Sections for Round/Square Pipes (mm)
Internal grid bars	20/2.01 20/2.09 9/2.83 12/2.86 4/2.94	65	88.9 × 3.2/ 70 × 70 × 3
External grid bars	16/2.24	16	70.0 × 3.2/ 60 × 60 × 3
Grid support bars	4/2.24	4	124.4 × 4.0/ 100 × 100 × 5
Branches	4/1.65 2/2.44 2/2.99	8	38 × 3.2/ 40 × 40 × 3
Columns	2/3.58	2	54 × 3.2/ 50 × 50 × 3

## Data Availability

Data are contained within the article.

## Appendix A

**Table A1:** Structural analysis results of structure 1a performed by Robot Structural Analysis Professional software.

Kind of the Structural Element	Bars’ Radius/Wall Thickness (mm)	Ratio (%)
Internal grid bars	57.0/3.2	91
External grid bars Grid’s support bars Branches Columns	76.1/3.2 10.8/4.0 30.0/3.2 57.0/3.2	83 90 76 87

Total mas 1506.14 kg.

**Table A2:** Structural analysis results of structure 3a performed by Robot Structural Analysis Professional software.

Kind of the Structural Element	Bars’ Radius/Wall Thickness (mm)	Ratio (%)
Internal grid bars	60.3/3.2	96
External grid bars Grid’s support bars Branches Columns	57.0/3.2 88.9/3.6 30.0/3.2 60.3/3.2	92 96 89 92

Total mas 1473.55 kg.

**Table A3:** Structural analysis results of structure 1b performed by Robot Structural Analysis Professional software.

Kind of the Structural. Element	Bars’ Radius/Wall Thickness (mm)	Ratio (%)
Internal grid bars	60.3/3.2	97
External grid bars Grid’s support bars Branches Columns	54.0/3.2 88.9/3.6 30.0/3.2 60.3/3.2	99 97 91 93

Total mas 1465.50 kg.

**Table A4:** Structural analysis results of structure 3b performed by Robot Structural Analysis Professional software.

Kind of the Structural Element	Bars’ Radius/Wall Thickness (mm)	Ratio (%)
Internal grid bars	60.3/3.2	93
External grid bars Grid’s support bars Branches Columns	48.3/3.2 88.9/3.6 30.0/3.2 54.0/3.2	96 94 75 73

Total mas 1451.91 kg.
